# Convex Regular Polychora Nanocrystals with Dipole–Dipole Interactions

**DOI:** 10.3390/nano15100771

**Published:** 2025-05-21

**Authors:** Orion Ciftja, Josep Batle, Mohamed Ahmed Hafez

**Affiliations:** 1 Department of Physics, Prairie View A&M University, Prairie View, TX 77446, USA; 2 Departament de Física and Institut d’Aplicacions Computacionals de Codi Comunitari (IAC3), University of the Balearic Islands, E-07122 Palma de Mallorca, Spain; jbv276@uib.es; 3 CRISP—Centre de Recerca Independent de sa Pobla, E-07420 Sa Pobla, Spain; 4 Department of Civil Engineering, Faculty of Engineering, INTI International University, Nilai 71800, Malaysia; mohdahmed.hafez@newinti.edu.my

**Keywords:** dipole–dipole interaction, equilibrium configuration, Platonic solids, regular polychora

## Abstract

Structures composed of classical dipoles in higher-dimensional space present a unique opportunity to venture beyond the conventional paradigm of few-body or cluster physics. In this work, we consider the six convex regular polychora that exist in an Euclidean four-dimensional space as a theoretical benchmark for hte investigation of dipolar systems in higher dimensions. The structures under consideration represent the four-dimensional counterparts of the well-known Platonic solids in three-dimensions. A dipole is placed in each vertex of the structure and is allowed to interact with the rest of the system via the usual dipole–dipole interaction generalized to the higher dimension. We use numerical tools to minimize the total interaction energy of the systems and observe that all six structures represent dipole clusters with a zero net dipole moment. The minimum energy is achieved for dipoles arranging themselves with orientations whose angles are commensurate or irrational fractions of the number π.

## 1. Introduction

In a three-dimensional (3D) space, the regular polyhedra, also known as Platonic solids, are a special class of polyhedra that have the following characteristics. (i) Identical faces: All faces of a regular polyhedron are congruent regular polygons (polygons where all sides and angles are equal). (ii) Identical vertices: Each vertex has the same number of edges meeting at it, making the polyhedron vertex-transitive. (iii) Symmetry: Regular polyhedra have high symmetry, meaning they look the same after being rotated or reflected in space. There are exactly five regular polyhedra in 3D space, the tetrahedron, cube, octahedron, dodecahedron, and icosahedron. Each has its own distinct properties. For instance, a tetrahedron has four triangular faces, four vertices and six edges. If a particle is localized at each vertex, the resulting structure may be viewed as a nanocrystal containing a small finite number of particles. Depending on the nature of interaction between the particles, the nanocrystal may often exhibit unique properties that differ from those of bulk materials. These properties arise because a significant proportion of the particles are located at or near the surface, where they behave differently from those in the interior. Undoubtedly, the five regular polyhedra predate human history. They appear in many natural forms and have emerged early in mathematical history. These five are the only regular polyhedra as confirmed by multiple proofs, for example, via the classical approach analyzing permissible face polygons and vertex angle sums, the topological proof leveraging the Euler characteristic, and Legendre’s elegant spherical geometry argument. A less refined but dimensionally generalizable proof examines the ratio of edge length to circumscribed sphere diameter, connecting it to the corresponding ratio of the lower-dimensional vertex figure (the convex hull of a vertex’s neighboring vertices).

In any *d*-dimensional space, $R^{d}$, generalizations of the tetrahedron, cube, and octahedron exist. They are known as the *d*-simplex, *d*-cube, and *d*-orthoplex. Beyond these, the only regular polytopes are the dodecahedron and icosahedron in $R^{3}$ and three exceptional polytopes in $R^{4}$: the 24-cell, 120-cell, and 600-cell, named for their respective facet counts. The 120-cell and 600-cell are duals. Each $R^{d}$ also admits a generalized cube tiling, the *d*-cube tiling. The sole non-cube regular tilings are two in $R^{2}$ (the dual triangle and hexagon tilings) and a pair of dual tilings in $R^{4}$ using 24-cells and 4-orthoplexes. The interest in these high-dimensional objects is motivated by the appealing underlying beauty, cemented by an inherent sense of symmetry. The broad concept of symmetry (its preservation or breaking) is to be found not only in mathematical structures, but also at the heart of physics. In the present work, we extend this curiosity to regular structures that are found in d=4 dimensions. The systems that we consider are the only six polytopes that are regular in a four-dimensional (4D) space [1] consisting of classical dipoles located at their vertices.

Systems with dipole–dipole interactions are important because they play a crucial role in determining the physical properties of many materials, especially in molecular and condensed matter systems [2,3,4,5,6,7,8]. Dipole systems in one-dimensional (1D) arrays are relatively simple to study. Having the dipoles interact in a 1D linear arrangement leads to more predictable and analytically tractable behavior [9,10]. In higher dimensions, however, the anisotropic and long-range nature of dipole–dipole interactions leads to frustration, complex ordering, and richer phase behavior, making the systems much more challenging to analyze [11,12]. These interactions influence phase behavior, self-assembly, and structural organization in soft matter, such as liquid crystals and biological membranes. Additionally, dipole–dipole interactions are fundamental in areas like quantum computing and molecular spectroscopy, where they affect energy transfer and coherence in quantum systems.

Significant experimental and theoretical advancements have been made in recent years concerning the characterization of dipolar gases with large dipole moments [13,14,15]. At sufficiently low temperatures, the formation of a classical crystal composed of dipolar particles becomes a plausible scenario. However, given the intrinsic nature of the dipole–dipole interaction, such a configuration is only viable if this force remains stable against thermal fluctuations. Systems involving dipoles oriented in different planes have been previously studied, both from a classical perspective [16,17,18,19,20] and a quantum mechanical one [21,22,23,24,25,26]. A key observation is that, due to various quantum effects, dipolar interactions exhibit unconventional properties in helium [27] and can also explain certain phenomena observed in magnetic colloids [28,29]. In most physical systems, the dipole–dipole interaction is relatively weak compared to other forces governing structural stability and material ordering. Nevertheless, this interaction is crucial in shaping magnetic domain orders. Therefore, to ensure the mathematical formalism is physically meaningful and amenable to classical treatment, dipole moments must be considered large.

## 2. Exploration of the Minimum Energy and Equilibrium Configurations

**Figure 1:**
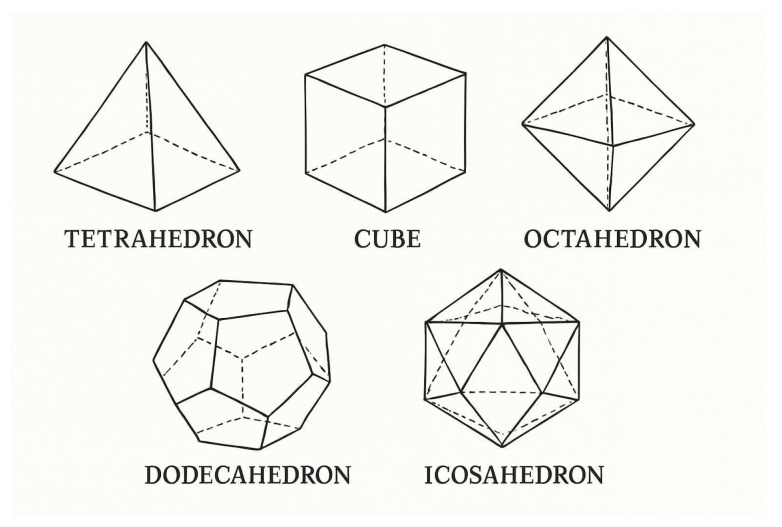
(Color online) Schematic wireframe diagrams of the five Platonic solids in 3D space. These solids are generalized to 4D space in the form of the regular polychora, a set of six 4D convex bodies (one more with respect to the Platonic ones). See text for details.

As already mentioned, there are only five regular polyhedra (Platonic solids) in a 3D space, as shown in Figure 1. In a 4D space, the number of possible regular polychora (4D analogs) is limited to six by the constraints of geometric regularity. A regular polychoron in a 4D space must consist of identical regular polyhedral cells meeting in identical configurations around each edge and vertex. The key restriction arises from the Schläfli symbol {p,q,r} [1]. This symbol is a notation used in geometry to describe a regular polychoron with *p*-sided polygonal faces, *q* such faces around each vertex in the cell (a regular polyhedron denoted as {p,q} represents the 3D cell) and *r* such cells around each edge. The condition that the dihedral angles of the cells must sum to less than $360^{\circ }$ around an edge (to avoid overlap) limits the possible combinations of {p,q,r}. Only six sets of integers satisfy this and other geometric constraints in 4D space: the 5-cell ({3,3,3}), 8-cell ({4,3,3}), 16-cell ({3,3,4}), 24-cell ({3,4,3}), 120-cell ({5,3,3}), and 600-cell ({3,3,5}). The reasoning mirrors the 3D case but extends to higher-dimensional symmetry. For instance, just as the icosahedron ({3,5}) cannot tile 3D space (as five tetrahedra around an edge leave a gap, but six overlap), only certain configurations close neatly in 4D space. The 24-cell ({3,4,3}) is unique to 4D space arising from the exceptional symmetry of octahedral cells ({3,4}) fitting four around each edge. The other polychora correspond to 4D analogs of the tetrahedron, cube, and dodecahedron. The nonexistence of a seventh regular polychoron follows from the fact that any other {p,q,r} would either fail to close properly or exceed angle bounds, just as attempting to construct a Platonic solid with hexagonal faces fails in a 3D scenario. Therefore, only six regular polychora exist as witnesses to the elegant interplay of geometry and dimensionality.

The objective of our work is to study a system consisting of identical dipoles each with a fixed dipole moment μ in an arbitrary spatial dimension (in our case, a 4D space). Unless otherwise noted, vectors are represented by bold symbols (e.g., v or μ). In some cases, particularly for emphasis or clarity, vectors may instead be written with an arrow overhead, as in $\vec{v}$ or $\vec{r}$. Both styles indicate vectors. The 4D dipole moment vector, $\mu \equiv \vec{\mu }$ is then represented as follows:

(1) $$\begin{array}{c} \mu =\mu \left(\begin{array}{c} \sin\left(\theta \right)\sin\left(\phi_{1}\right)\cos\left(\phi_{2}\right)\\ \sin\left(\theta \right)\sin\left(\phi_{1}\right)\sin\left(\phi_{2}\right)\\ \sin\left(\theta \right)\cos\left(\phi_{1}\right)\\ \cos(\theta ) \end{array}\right)\;. \end{array}$$

The interaction energy between any two dipoles, $\mu_{u}$ and $\mu_{v}$ localized at positions $r_{u}$ and $r_{v}$, respectively, is given by

(2) $$E_{u,v}=C\left(\frac{\mu_{u}\cdot \mu_{v}}{r_{uv}^{3}}-\frac{3\left(\mu_{u}\cdot r_{uv}\right)\left(\mu_{v}\cdot r_{uv}\right)}{r_{uv}^{5}}\right)\;,$$

where $r_{uv}$ is the vector between the positions of the two dipoles *u* and *v* and $r_{uv}=\left|r_{uv}\right|\geq 0$ is the corresponding separation distance. The constant *C* is either $\frac{\mu_{0}}{4\pi }$ (for magnetic dipoles) or $\frac{1}{4\pi \epsilon_{0}}$ (for electric dipoles).

Physically, the classical extremum energy states (either minimum or maximum value of energy) of a magnetic dipole system correspond to one of equilibrium in which no torque should act on any given dipole. Let us now consider the entire system of *N* dipoles. By slightly changing the notation, we write the general Hamiltonian as follows:

(3) $$H=\sum_{k,i,l,j}\sum_{\alpha ,\beta }\mu_{i}^{\alpha }\left(R^{k}\right)J_{i\;j}^{\alpha \;\beta }\left(R_{ij}^{kl}\right)\mu_{j}^{\beta }\left(R^{l}\right)\;,$$

where

(4) $$\begin{array}{ccc} J_{i\;j}^{\alpha \;\beta }\left(R\right) & = & \frac{D}{2}\;\left(\frac{\delta_{\alpha \beta }}{{\left|R\right|}^{3}}-3\frac{R^{\alpha }R^{\beta }}{{\left|R\right|}^{5}}\right)\;. \end{array}$$

Here, $R_{ij}^{kl}$ represents the separation vector between two classical O(d) spins, $\mu_{i}^{\alpha }\left(R^{k}\right)$ and $\mu_{j}^{\beta }\left(R^{l}\right)$, both of unit length. The indices *k* and *l* label the unit cells, while *i* and *j* enumerate the basis sites within the unit cell. Greek indices α and β indicate the vector components (x,y,z,…). The prefactor $\frac{1}{2}$ accounts for the avoidance of double counting. The constants *C* and $D=\mu^{2}\;C$ encode the (electric or magnetic) nature of the dipoles. For our purposes, we will suppress these terms (we set them to one) and present all energies in dimensionless units. We remark that the Hamiltonian in Equation (3) admits an elegant mathematical formulation as a quadratic form.

Now that the Hamiltonian of the system is defined, we next require a proper parametrization for the orientation of the dipole moment in O(d). We shall take the usual extension of spherical coordinates for all dipoles in the following form:

(5) $$\begin{array}{ccc} x_{1}\; & = & \;r\;\sin\left(\psi_{1}\right)\cdots \sin\left(\psi_{n-2}\right)\cos\left(\psi_{n-1}\right)\\ x_{2}\; & = & \;r\;\sin\left(\psi_{1}\right)\cdots \sin\left(\psi_{n-2}\right)\sin\left(\psi_{n-1}\right)\\ x_{3}\; & = & \;r\;\sin\left(\psi_{1}\right)\cdots \cos\left(\psi_{n-2}\right)\\ & \vdots & \\ x_{n-2}\; & = & \;r\;\sin\left(\psi_{1}\right)\sin\left(\psi_{2}\right)\sin\left(\psi_{3}\right)\\ x_{n-1}\; & = & \;r\;\sin\left(\psi_{1}\right)\cos\left(\psi_{2}\right)\\ x_{n}\; & = & \;r\;\cos(\psi_{1})\;. \end{array}$$

In the notation of Equation (5), $x_{i}$ denotes components of the dipole moment vector while *r* is its corresponding magnitude. Within the framework of the dipole–dipole interaction, the magnitude of the dipole moment is not necessary for defining its direction. Therefore, we shall employ unit dipoles, and we shall have r=1 in Equation (5). From Equation (5), we observe that a dipole in *d* dimensions requires a set of d−1 independent angles $\left\{\psi_{1},\psi_{2},\cdots ,\psi_{d-1}\right\}$ to fully define its orientation in O(d). Depending on the polytope, the number of vertices *V* will grow following different rates. However, the total number of variables to employ in the minimization of Equation (3) will become considerable with increasing *d* once the positions of the *V* vertices $R_{k}$ are given.

Consequently, practical analysis typically requires approximate or heuristic approaches. The most effective statistical method currently available is Kirkpatrick, Gelatt, and Vecchi’s simulated annealing approach [30], which implements the Metropolis Monte Carlo algorithm with a constant temperature at each stage of the annealing process. Alternative non-statistical approaches include downhill/amoeba and gradient methods [31]. These techniques employ finite differences when evaluating the objective function across all relevant real variables. In our case, it shall suffice to employ the simulated method throughout our computations. Regarding the position vectors $R_{k}$, we shall choose the standard definition of the coordinates of the vertices in all three polytopes, centered at the origin, and tailored to have unit edge length. Three classes of regular polytopes exist for all dimensions [1]. These are the *d*-simplex, the *d*-orthoplex or cross-polytope, and the *d*-cube. For the d=4 case, there are six of them, which we study separately in each of the following six subsections (Section 2.1, Section 2.2, Section 2.3, Section 2.4, Section 2.5 and Section 2.6).

### 2.1. The 5-Cell (4-Simplex)

The unit length d=4-simplex, $T_{\Delta }$ has its vertices given by the *N* vectors (N=d+1):

(6) $$\begin{array}{ccc} \vec{r_{1}} & = & \left(-\frac{1}{2},-\frac{1}{2\sqrt{3}},-\frac{1}{4}\sqrt{\frac{2}{3}},\ldots ,-\frac{1}{N-1}\sqrt{\frac{N-1}{2N}}\right)\\ \vec{r_{2}} & = & \left(\frac{1}{2},-\frac{1}{2\sqrt{3}},-\frac{1}{4}\sqrt{\frac{2}{3}},\ldots ,-\frac{1}{N-1}\sqrt{\frac{N-1}{2N}}\right)\\ \vec{r_{3}} & = & \left(0,\frac{1}{\sqrt{3}},-\frac{1}{4}\sqrt{\frac{2}{3}},\ldots ,-\frac{1}{N-1}\sqrt{\frac{N-1}{2N}}\right)\\ \vec{r_{4}} & = & \left(0,0,\frac{3}{4}\sqrt{\frac{2}{3}},\ldots ,-\frac{1}{N-1}\sqrt{\frac{N-1}{2N}}\right)\\ \ldots & & \\ \vec{r_{N}} & = & (0,0,0,\ldots ,\sqrt{\frac{N-1}{2N}})\;, \end{array}$$

where the quantity $\sqrt{\frac{N-1}{2N}}$ gives the center-to-vertex distance for a regular *N*-polytope of unit edge length. It is straightforward to verify that this configuration satisfies $|\sum_{i}\vec{r_{i}}{|}^{2}=\sum_{i,\;j}\vec{r_{i}}\cdot \vec{r_{j}}=0$ as demanded by the geometric constraints. This specific vertex arrangement for the *N*-simplex offers the computational advantage of simplifying dimensional expansion. This way, each new dimension requires only the addition of a fresh azimuthal axis to the existing structure. These vectors also comply with the relation $\vec{r_{i}}\cdot \vec{r_{j}}\;=\;-\frac{1}{2N}+\frac{1}{2}\delta_{ij}$.

The 5-cell is the simplest of the 4D structures under current scrutiny, with the number of vertices being V=5 and number of edges being E=10. The minimum energy is found to be $E_{\min}=-6.25$ exactly and $|\sum_{k}\mu_{k}|=0$. The values for various parameters that correspond to the minimum total energy configuration are shown in Table 1. We found that the scalar product, $\mu_{i}\cdot \mu_{j}$ is the same for all dipoles, whereas the energy contributions of the interacting dipoles are defined by a finite set of different values for all pairs. In a nutshell, their sum adds up to a rational number. An interesting result is that $R_{k}\cdot \mu_{k}=0$ for all *k*. That is, all dipoles are perpendicular the their vector positions. The corresponding equilibrium angles are given in Table 2. As in the case of the more familiar 3-simplex (tetrahedron) they are irrational fractions of number, π.

### 2.2. The 5-Cell (4-Orthoplex)

Orthoplexes or cross-polytopes are regular, convex polytopes that extend the regular octahedron to higher dimensions. Going from one dimension to the next one adds two vertices each time. As opposed to the simplex, one more pairwise distance is added, $\sqrt{2}$. Specifically, all these dipoles that are furthest away contribute to the energy only with $\mu_{i}\cdot \mu_{j}=-1$, in all dimensions. For this regular polytope, the number of vertices is V=8 and the number of edges is E=24. We obtain a minimum energy, $E_{\min}=-13.4142085$ and $|\sum_{k}\mu_{k}|=0$. The values for various parameters that correspond to the minimum total energy configuration for the case of a 5-cell (4-orthoplex) are shown in Table 3.

We also found that $R_{k}\cdot \mu_{k}=0$ for all *k*. The values of the equilibrium angles for the case of a 5-cell (4-orthoplex) are shown in Table 4. We also investigated the possibility of obtaining an analytic expression for the minimum energy. We found that the precise expression for the components of the dipole moments are given as $\{{\left(0,0,a,b\right)}_{1},{\left(0,0,-b,a\right)}_{2},$${\left(-c,d,0,e\right)}_{3}$$,{\left(-d,-c,-e,0\right)}_{4},{\left(0,0,-a,-b\right)}_{5},$${\left(0,0,b,-a\right)}_{6},$${\left(c,-d,0,-e\right)}_{7},{\left(d,c,e,0\right)}_{8}\}$, with $a^{2}+b^{2}=c^{2}+d^{2}+e^{2}=1,\;0<a,c,d<1$. To the best of our knowledge, the solution to the corresponding minimization procedure returns an expression for $E_{\min}$ that seems to be given in a transcendental form.

### 2.3. The 8-Cell (Hypercube)

Let us recall that the 8-cell (also known as the tesseract or hypercube) is a body formed by the convex hull of points $\left(\pm \frac{1}{2},\pm \frac{1}{2},\ldots ,\pm \frac{1}{2},\pm \frac{1}{2}\right)$ in *d* dimensions (the number of vertices for such a case would be $V=2^{d}$). This situation has been partially studied in ref. [32]. For the 3D cube, the equilibrium angles are given in terms of two incommensurate numbers, namely, $\theta_{c}=39.648251^{\circ }$ and $\phi_{c}=76.429636^{\circ }$. The cube constitutes one of the few instances where one can actually notice the transcendental nature of the equilibrium angles. Incidentally, the cube (and the tetrahedron) are the only instances among all five regular polyhedra where irrational numbers occur. Extending the previous calculations to the tesseract (hypercube), one obtains the value of energy, $E_{\min}\;=\;-\;30.975117$. The surprising outcome is that the tesseract (hypercube), which possesses a total null dipole moment as well, supports commensurate equilibrium angles. As we shall see, out of the three angles $\left(\psi_{1},\psi_{2},\psi_{3}\right)$ for the dipole orientations, the third angle in each case can be arbitrary. It is known that d−cubes present a richer structure than the other two families of regular polytopes. The representation chosen (construction and enumeration) is such that we enumerate the vertices k=1,…,16 following the binary representation {0000,0001,0010,0011,⋯,1110,1111} and then replacing “0” with “$-\frac{1}{2}$”, “1” with “$\frac{1}{2}$” and so on. In this fashion we guarantee that the vector sum of all positions is null and that the *d*-cube is centered at the origin.

The 4-cube or tesseract (hypercube) has 16 vertices, 32 edges, 24 square faces, and 8 cubes (hence the name “8-cell”). Thus, metrically speaking, we shall encounter edges of length 1, square diagonals of length $\sqrt{2}$, opposite cube vertices with length $\sqrt{3}$, and the diagonals of the 4-cube of length 2 (the maximum length in an *d*-cube is $\sqrt{d}$). The corresponding equilibrium angles ($\psi_{3}$ arbitrary) for the case of the 8-cell (hypercube) are shown in Table 5. At further scrutiny, we can conclude that all angles involved in the dipole orientations are commensurate.

### 2.4. The 24-Cell

**Figure 2:**
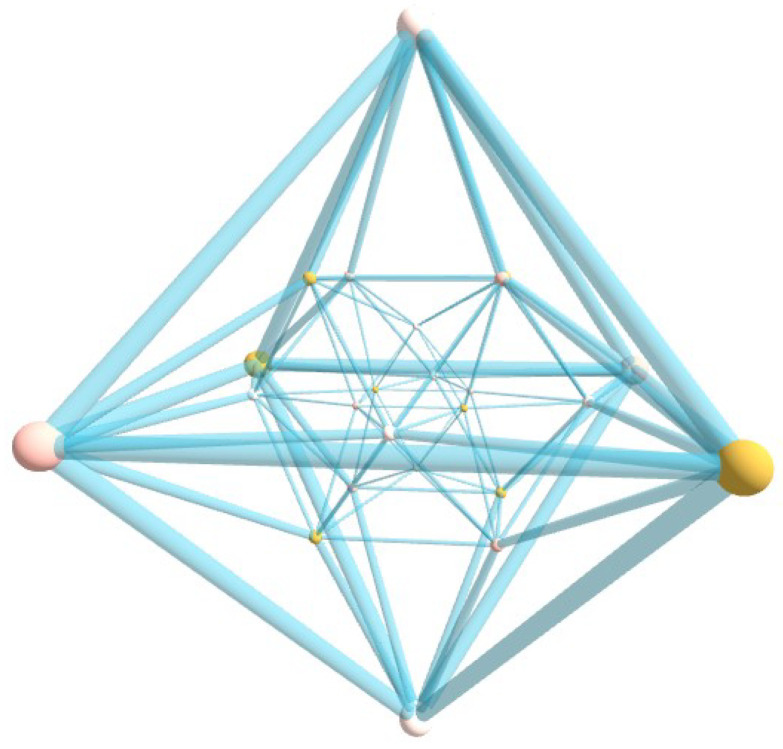
(Color online) Projection of the 24-cell into $R^{3}$. The polychoron is made of 24 regular octahedra joined at 96 triangular faces, with three around each edge. See text for details.

A visual perspective of the 24-cell polychoron is provided in Figure 2 where we show its projection in 3D space.

The 24-cell does not have a regular analogue in 3D space or any other number of dimensions. Thus, it is the only one of the six convex regular polychora in 4D which is not the analogue of one of the five Platonic solids in 3D. It has V=24 vertices and E=96 edges. Numerical calculations show that the mininum energy is $E_{\min}=-52.0843285$ and $|\sum_{k}\mu_{k}|=0$. The values of the equilibrium angles are shown in Table 6.

Some angles follow a certain pattern. For instance, we can find the relations for $\psi_{1}$ such as 60.3279675+119.672036=70.1473413+109.852657=180.

### 2.5. The 120-Cell

**Figure 3:**
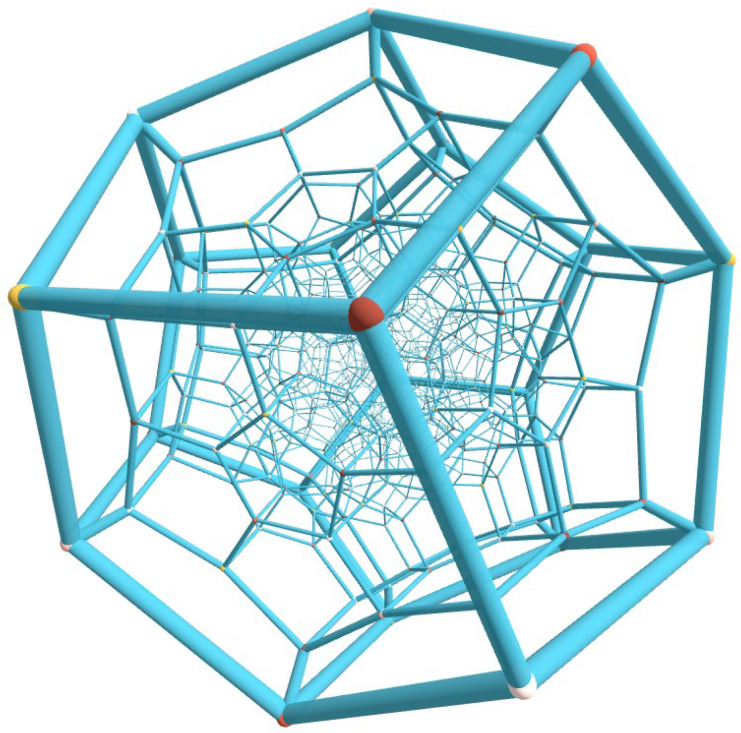
(Color online) Projection of the 120-cell into $R^{3}$. The 120-cell is the 4D analogue of the dodecahedron. It consists of 120 dodecahedra joined at 720 faces, with three dodecahedra around each edge. See text for details.

Projection of the 120-cell into $R^{3}$ is shown in Figure 3. The 120-cell is the 4D analogue of the dodecahedron. It consists of 120 regular dodecahedra joined at 720 regular pentagon faces, with three dodecahedra around each edge. It has V=600 vertices and E=1200 edges.

The regular 120-cell polytope in $R^{4}$ represents one of the most exquisite structures in mathematics. Its surface comprises 120 regular dodecahedral cells, making this configuration exceptionally rare as it naturally exists in three distinct mathematical domains: as a regular polytope in $R^{4}$, embedded in the remarkable sphere $S^{3}$, and within the quaternion space H. Notably, the 120-cell encapsulates both the icosahedral symmetry and the topological structure of the Poincaré homology sphere. Through extensive numerical minimization (involving 600×3=1800 degrees of freedom), we obtained the following optimal values for various quantities of the interacting system: $E_{\min}=-2837.78743$, $|\sum_{k}\mu_{k}|=0.5600865$, and $\sum_{k}R_{k}\cdot \mu_{k}=0.317436$. These results represent the lowest energy configuration achieved without imposing constraints on either $|\sum_{k}\mu_{k}|$ or $\sum_{k}R_{k}\cdot \mu_{k}$. Note that the 120-cell is arguably the most complex of the six regular 4D polytopes. Therefore, even if the net moment is not exactly zero, its value ($|\sum_{k}\mu_{k}|=0.5600865$) can be considered practically zero. Due to limited computational resources and the numerical difficulties of handling this challenging case study, we would tend to believe that $|\sum_{k}\mu_{k}|=0$ for all practical purposes. We noticed that when constraints are imposed during the optimization process, one can obtain a vanishing net moment but at the expense of a slightly higher energy. Overall, the numerical results, while computationally challenging, seem to suggest that the 120-cell configuration is not a polar molecular arrangement but rather features dipoles oriented perpendicular to their position vectors. Central projection of the 120-cell onto its circumscribed $S^{3}$ generates a tessellation by congruent regular spherical dodecahedra. The stereographic projection preserves both spherical surfaces and angular relationships, yielding a partition of $R^{3}$ into 120 spherical domains. These domains exhibit remarkable geometric properties: adjacent spherical surfaces intersect at $120^{\circ }$ along edges, with four surfaces meeting at each vertex—a configuration analogous to soap bubble clusters and mathematically established by J. Taylor’s theorem [33]. The values of the equilibrium angles are shown in Table 7.

### 2.6. The 600-Cell

**Figure 4:**
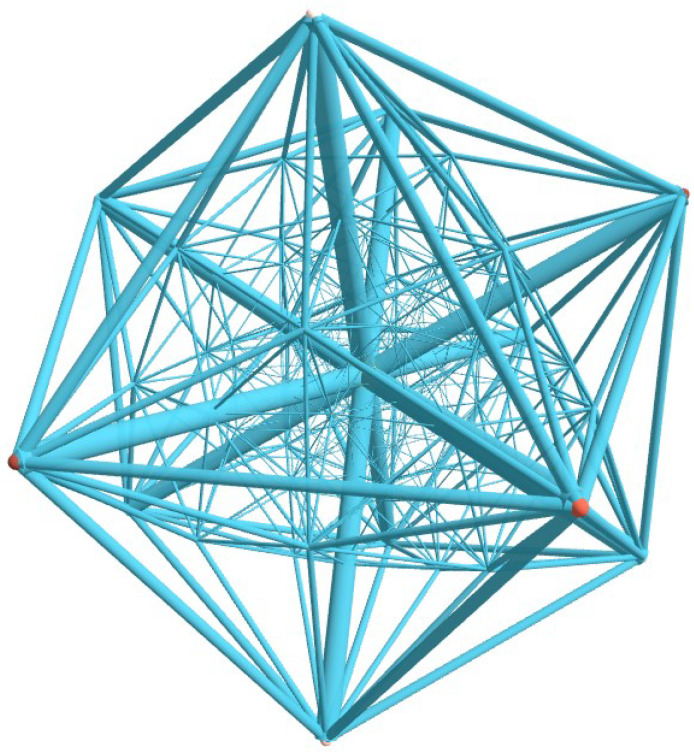
(Color online) Projection of the 600-cell into $R^{3}$. The 600-cell is the 4D analogue of the icosahedron. It consists of 600 tetrahedra joined at 1200 faces with five tetrahedra around each edge. Its 120 vertices can be partitioned into five sets which form the vertices of five inscribed 24-cells. See text for details.

The 600-cell structure concludes our study. Projection of the 600-cell into 3D space is shown in Figure 4. The 600-cell is the 4D analogue of the icosahedron. It consists of 600 regular tetrahedra joined at 1200 triangular faces with five tetrahedra around each edge. The number of vertices is V=120. Its 120 vertices can be partitioned into five sets, which form the vertices of five inscribed 24-cells.

Numerical calculation of the mininum energy resulted in $E_{\min}=-342.09652$ and $|\sum_{k}\mu_{k}|=0$. The 600-cell is an object where analogies have been found in soap bubbles as well as other areas of physics. For instance, the system of 60 rays derived from the vertices of a 600-cell is used to provide proofs of the Bell–Kochen–Specker theorem, which rules out the existence of non-contextual hidden variables theories [34].

## 3. Conclusions

Regular polychora are 4D analogs of the regular polyhedra, consisting of cells that are identical regular polyhedra arranged in a symmetrical, 4D structure. There are exactly six convex regular polychora each exhibiting uniformity in vertices, edges, faces, and cells. The six convex regular polychora (also known as the regular 4-polytopes) that we study in this work are as follows: (i) 5-cell (4-simplex) made of 5 tetrahedral cells; (ii) 8-cell (tesseract or hypercube) made of 8 cubic cells; (iii) 16-cell made of 16 tetrahedral cells; (iv) 24-cell made of 24 octahedral cells (unique to 4D space); (v) 120-cell made of 120 dodecahedral cells; and (vi) 600-cell made of 600 tetrahedral cells. These are the 4D analogs of the familiar Platonic solids, exhibiting perfect symmetry and regularity. The system under consideration consists of 4D dipoles placed in each vertex of the 4D structure under consideration. The dipoles are allowed to interact with the rest of the system. We numerically minimize the total interaction energy of all the systems under consideration. This way, we identify the minimum energy configurations of dipoles for the six structures that conform to the convex regular polychora. We observe that the minimum energy configuration corresponds to clusters of dipoles with a zero net dipole moment. The dipoles arrange themselves in orientations whose angles are commensurate or irrational fractions of number, π.

Our detailed study reveals one important consequence of the interplay between the high degree of symmetry of these polytopes and the results of considering a physical approach. For all interacting dipoles, we do encounter that all of them *are not polar* clusters. That is, the vector sum of all dipoles, $|\sum_{k}\mu_{k}|$ turns out to be zero. This result is indeed remarkable. Having zero total magnetic moment is a condition that can be achieved in many ways. Among various options, there is only one configuration up to trivial rotations, which has the lowest minimum energy possible. We hope that this contribution will inspire further research connecting high-dimensional mathematical structures with physical phenomena involving dipolar systems based on the rich array of characteristics that define regular polytopes from the perspective of geometrical symmetry. 

## Figures and Tables

**Table 1:** Values of parameters corresponding to the configuration with minimum total energy for the case of a 5-cell (4-simplex).

pairi,j	$|R_{\mathrm{ij}}|$	$\mu_{i}\cdot \mu_{j}$	$R_{\mathrm{ij}}\cdot \mu_{i}$	$R_{\mathrm{ij}}\cdot \mu_{j}$
1–2	1.	−1/4	0.0115152961	0.0115153044
1–3	1.	−1/4	0.546703935	0.546703935
1–4	1.	−1/4	−0.429405749	−0.429405779
1–5	1.	−1/4	−0.128813446	−0.12881346
2–3	1.	−1/4	0.0342390686	0.0342390686
2–4	1.	−1/4	0.487855941	0.487855941
2–5	1.	−1/4	−0.510579705	−0.510579705
3–4	1.	−1/4	0.165581286	0.165581286
3–5	1.	−1/4	0.415361702	0.415361702
4–5	1.	−1/4	0.224031493	0.224031493

**Table 2:** Values of equilibrium angles corresponding to the configuration with minimum total energy for the case of a 5-cell (4-simplex).

#	$\psi_{1}$	$\psi_{2}$	$\psi_{3}$
1	72.506818	178.870271	90
2	176.794781	19.6527469	14.4774901
3	104.464518	69.7335025	197.894282
4	77.0540669	70.7978859	317.535889
5	77.0540669	68.9360805	77.0589669

**Table 3:** Values of parameters corresponding to the configuration with minimum total energy for the case of a 5-cell (4-orthoplex).

pairi,j	$|R_{\mathrm{ij}}|$	$\mu_{i}\cdot \mu_{j}$	$R_{\mathrm{ij}}\cdot \mu_{i}$	$R_{\mathrm{ij}}\cdot \mu_{j}$
1–2	1.	0.	0.	0.
1–3	1.	0.0155619718	0.65871948	0.658123016
1–4	1.	−0.0397607274	0.257081866	0.256853014
1–5	1.41421356	−1.	0.	0.
1–6	1.	0.	0.	0.
1–7	1.	−0.0155245336	−0.658716857	−0.658115387
1–8	1.	0.0398687907	−0.257079214	−0.256854534
2–3	1.	0.0398293436	−0.257105738	−0.25684154
2–4	1.	0.0155253429	0.658709109	0.6581195
2–5	1.	0.	0.	0.
2–6	1.	−1.	0.	0.
2–7	1.	−0.0397706963	0.257108361	0.256851226
2–8	1.	−0.0155527722	−0.658706486	−0.658120155
3–4	1.	0.	0.0302283186	0.0301849879
3–5	1.	−0.0155616254	0.658103526	0.658714354
3–6	1.	−0.0398307964	−0.256861061	−0.257066846
3–7	1.41421356	−1.	0.	0.
3–8	1.	0.	−0.030247841	−0.0302667264
4–5	1.	0.0397592485	0.256846815	0.257086128
4–6	1.	−0.0155230397	0.658113301	0.658722699
4–7	1.	0.	0.0301787779	0.0301900022
4–8	1.41421356	−1.	0.	0.
5–6	1.	0.	0.	0.
5–7	1.	0.0155241862	0.658716679	0.658115745
5–8	1.	−0.039867308	0.257088453	0.256841332
6–7	1.	0.0397721492	−0.257064193	−0.256850868
6–8	1.	0.0155504625	0.658725321	0.658106983
7–8	1.	0.	0.0301896483	0.0302535053

**Table 4:** Values of equilibrium angles corresponding to the configuration with minimum total energy for the case of a 5-cell (4-orthoplex).

#	$\psi_{1}$	$\psi_{2}$	$\psi_{3}$
1	111.319407	0.000117426552	236.903599
2	158.678288	180.	-
3	87.5491073	90.	158.680078
4	90.	92.446335	68.6802738
5	68.6800075	180.	-
6	21.3180991	180.	-
7	87.553021	90.	158.680164
8	90.	92.4526797	68.6803405

**Table 5:** Values of equilibrium angles corresponding to the configuration with minimum total energy for the case of an 8-cell (hypercube).

	$\psi_{2}=0$	$\psi_{2}=180-0$
$\psi_{1}=45$	6, 8, 9, 12, 15	1, 2, 4, 5, 10, 11
$\psi_{1}=180-45$	14, 16	3, 7, 13

**Table 6:** Values of equilibrium angles corresponding to the configuration with minimum total energy for the case of a 24-cell polychoron.

#	$\psi_{1}$	$\psi_{2}$	$\psi_{3}$
1	119.672033	55.2676808	267.267396
2	119.672038	124.732322	177.267383
3	119.672036	124.732324	177.267382
4	60.3279675	55.2676807	87.2673897
5	109.852661	82.5847597	21.3525627
6	70.1473413	157.45348	289.66921
7	109.852657	157.453481	289.669206
8	109.852662	82.5847621	201.352561
9	146.578076	132.716264	67.4132491
10	98.941858	67.7751557	155.883178
11	98.9418568	112.224849	335.883174
12	33.4219267	132.716264	247.413261
13	44.5002174	92.7839139	135.
14	88.0491183	44.4663865	225.
15	91.9508813	135.533609	45.
16	44.5002147	87.2160917	135.
17	150.305603	133.278733	199.669186
18	83.0277066	110.006857	111.352558
19	83.0277055	69.9931407	291.352561
20	29.6944012	133.278732	19.6691968
21	68.0593955	154.132955	337.413249
22	68.0593944	99.6465005	65.883184
23	68.0593944	80.3535051	245.883186
24	68.0593927	154.132955	157.413254

**Table 7:** Values of equilibrium angles corresponding to the configuration with minimum total energy for the case of a 120-cell polytope.

#	$\psi_{1}$	$\psi_{2}$	$\psi_{3}$
1	30.3489433	132.124578	216.431719
2	30.3489497	53.3634847	236.708025
3	77.1411352	69.6580755	340.741152
4	102.858873	108.014987	291.440979
5	107.548319	25.1653819	326.70807
6	72.4516834	76.501923	21.440956
7	70.1898896	156.526716	306.43175
8	109.81011	76.3170357	250.741168
9	70.1898935	76.3170578	70.7411558
10	70.1898891	23.4732858	306.431726
11	107.548312	76.5019376	21.4409715
12	72.4516833	154.834603	326.708037
13	77.1411342	71.9850129	111.440976
14	77.1411292	110.341926	340.741166
15	30.3489466	126.636525	56.7080238
16	149.651052	47.8753938	36.4317228
17	58.3950084	48.7587873	90.0000134
18	121.604998	131.241217	269.99999
19	90.	50.1773151	226.972973
20	90.	50.1773253	46.972957
21	129.8227	90.	136.972953
22	129.822684	90.	316.972964
23	55.8435836	50.7060256	180.000006
24	124.156428	50.7060492	0.
25	25.4981031	116.517237	112.387436
26	120.451049	102.882294	305.979096
27	109.946665	40.3621803	35.8068957
28	86.8670893	135.834336	77.826069
29	93.1329047	135.834328	77.8260711
30	70.0533332	40.3621661	215.806905
31	59.5489739	102.8823	305.979115
32	154.501879	116.51727	112.387418
33	119.584444	65.8230269	244.532045
34	60.4155499	141.443997	20.7683572
35	98.4361966	68.896242	12.0207918
36	98.4361959	133.431583	274.36338
37	98.4361956	133.431585	94.3633624
38	81.5638158	68.896254	192.020784
39	60.4155543	38.5559931	20.7683572
40	60.4155606	65.8230332	64.5320302
41	36.9812093	96.1199211	267.324053
42	143.018782	40.1313209	139.758553
43	76.2681301	93.7853277	165.024353
44	76.2681391	61.7407756	315.711069
45	76.2681408	118.259229	315.711065
46	76.2681401	93.7853237	345.024367
47	143.018795	40.1313165	319.758577
48	143.018805	83.8800875	267.32409
49	49.2980146	82.1329146	216.118704
50	49.2980071	168.999064	124.806556
51	75.1477489	96.1627813	7.10012447
52	104.852241	140.345188	314.139832
53	104.852243	140.345188	314.139825
54	75.147766	83.8372308	187.100127
55	130.701994	11.0009432	304.806527
56	49.2980027	82.1329349	216.118696
57	47.1525101	132.329138	110.768367
58	132.847488	78.4570848	4.36335288
59	110.864689	58.1057473	154.532016
60	110.864674	99.0329975	282.020812
61	110.864696	80.9669647	102.020802
62	69.1353289	121.894238	334.532021
63	132.847498	101.542934	4.36336908
64	47.1525076	47.6708649	110.76841
65	63.7255992	118.624684	199.005876
66	63.7255854	47.4266575	80.9588614
67	96.8218944	16.1710649	247.960905
68	96.8218999	85.2294698	41.2280586
69	83.1780908	94.7705391	41.2280704
70	96.8218979	163.828942	247.96088
71	63.7255911	47.4266363	260.958852
72	63.7256009	61.3753313	19.0058687
73	20.5483461	135.529284	15.1183713
74	107.215264	74.7980084	150.068767
75	91.6001714	53.2942805	175.410068
76	52.2302812	139.098491	242.700823
77	127.769719	40.9015106	62.7007908
78	88.3998474	126.705765	355.410056
79	72.7847386	105.201974	150.068778
80	20.5483136	135.529278	195.118305
81	62.6174765	154.109672	49.7585694
82	62.6174919	74.4944718	45.7110548
83	93.6769652	143.176031	177.324073
84	93.6769717	103.760742	255.024354
85	86.323024	103.760759	255.024345
86	86.3230225	36.8239819	357.324068
87	117.382525	74.4944543	225.711068
88	117.382512	25.8903382	229.758588
89	162.484349	113.245639	337.960848
90	85.2633114	83.1546174	311.228052
91	127.346708	56.1629337	170.958874
92	115.440559	60.6459247	109.005871
93	115.440552	60.645934	109.005856
94	127.346724	123.837085	170.958861
95	85.2633235	96.8454027	311.228049
96	17.5156503	66.7543239	337.960903
97	44.25378	85.5082126	347.826043
98	135.74623	60.7342531	125.806885
99	78.9190513	23.1109157	202.387447
100	101.08095	58.9069123	215.97911
101	78.9190169	58.9069247	215.979098
102	101.080967	156.889082	202.387405
103	44.2537789	60.7342789	305.806903
104	44.2537602	85.5081573	347.826049
105	138.088763	12.5078992	214.806545
106	41.9112558	67.4346936	44.1398081
107	84.0438821	130.970036	126.118707
108	84.0438887	75.0652706	277.100124
109	84.0438928	75.0652713	277.100123
110	84.0438856	49.029971	126.11869
111	138.088767	67.4347136	44.1398124
112	41.911242	12.5079016	34.8065772
113	53.310918	40.1989522	332.700788
114	126.689081	88.0044022	85.4100821
115	75.4943983	165.28652	285.118357
116	75.4944024	72.199247	60.0687733
117	104.505611	107.800743	60.0687721
118	75.4943873	14.7134667	105.118352
119	126.689091	91.9956242	85.4100671
120	126.689083	40.1989414	332.700826

## Data Availability

Data is contained within the article.
